# The selective inhibition of the Syk tyrosine kinase ameliorates experimental autoimmune arthritis

**DOI:** 10.3389/fimmu.2023.1279155

**Published:** 2023-12-04

**Authors:** Eszter Káposztás, Lili Balogh, Attila Mócsai, Éva Kemecsei, Zoltán Jakus, Tamás Németh

**Affiliations:** ^1^ Department of Physiology, Semmelweis University School of Medicine, Budapest, Hungary; ^2^ MTA-SE “Lendület” Translational Rheumatology Research Group, Hungarian Academy of Sciences and Semmelweis University, Budapest, Hungary; ^3^ ELKH-SE Inflammation Physiology Research Group, Eötvös Loránd Research Network and Semmelweis University, Budapest, Hungary; ^4^ Department of Rheumatology and Clinical Immunology, Semmelweis University, Budapest, Hungary; ^5^ Department of Internal Medicine and Oncology, Semmelweis University, Budapest, Hungary

**Keywords:** rheumatoid arthritis, SYK (spleen tyrosine kinase), inhibitor, treatment, neutrophils

## Abstract

Autoimmune arthritis – such as rheumatoid arthritis – affect a significant proportion of the population, which can cause everyday joint pain, decreased mobility and reduced quality of life. Despite having more and more therapeutic options available, there are still a lot of patients who cannot reach remission or low disease activity by current therapies. This causes an urgent need for the development of new treatment options. The Syk tyrosine kinase plays an essential role in B cell receptor, Fc receptor and integrin signaling. It has been shown that the hematopoietic cell-specific deletion of Syk resulted in a complete protection against autoantibody-induced experimental arthritis. This prompted us to test the effect of entospletinib, a second generation, Syk-selective inhibitor, which has a tolerable safety profile according to hematological clinical trials, in experimental autoimmune arthritis. We found that entospletinib dose-dependently decreased the macroscopic signs of joint inflammation, while it did not affect the health status of the animals. In line with these findings, local neutrophil accumulation and cytokine levels were reduced compared to the vehicle-treated group, while macrophage accumulation and synovial fibroblast numbers were not significantly altered. Meanwhile, entospletinib dose-dependently decreased the cell responses of immune complex- or integrin ligand-activated neutrophils. Overall, we found that selective Syk inhibition by entospletinib reduced the activity of autoantibody-induced experimental arthritis, which seems to be based mainly on the effect of the inhibitor on neutrophil functions. Our data raise the possibility that entospletinib could be a good drug candidate in the treatment of human autoimmune arthritis.

## Introduction

Rheumatoid arthritis is a chronic systemic autoimmune disease, which affects approximately 1% of the total population and can lead to severe joint dysfunction and disability (1). Despite of the fact that there are more and more available drugs in the treatment (from conventional synthetic disease-modifying antirheumatic drugs to biological therapies or Janus-kinase inhibitors), there is still a significant proportion of patients who do not respond well to recent therapies (2). This causes an urgent need for the development and use of novel therapeutic agents in order to improve the quality of life of (difficult-to-treat) rheumatoid arthritis patients.

One important way to identify new therapeutic targets relies on a better understanding of the pathogenesis, for which animal models are useful. It has been previously shown that Fcγ receptors and integrins are essential in the development of an autoantibody-induced experimental arthritis model (3–5). The Syk tyrosine kinase is an important signaling component of various immune receptors (e.g. Fc receptors) and other cell surface molecules like integrins and plays essential roles in the activation of several immune cells (6–10). Due to the fact that many cell types (e.g. B cells, macrophages, neutrophils, synovial fibroblasts, etc.) that express Syk participate in the development and progression of rheumatoid arthritis, the molecule became a target in experimental arthritis in the mid 2000s (11). As R406, the active metabolite of the ‘so-called Syk-inhibitor’ fostamatinib (R788) was found to be effective in experimental arthritis models, clinical studies were initiated (12). While fostamatinib was found to be effective in the phase 2 clinical trial in patients with rheumatoid arthritis, it did not show significant efficacy over placebo in the phase 3 study, which led to the discontinuation of further investigation in the musculoskeletal field in Europe and North America (12–14). However, it turned out that fostamatinib is not a Syk-selective inhibitor, but rather a non-specific tyrosine kinase blocker, meaning that targeting Syk could be still an applicable pathway in the control of autoimmune arthritis (15). This was further strengthened by the finding that the hematopoietic cell-specific deletion of Syk resulted in a total protection against experimental autoantibody-induced arthritis, which was found to be (partly) based on the role of Syk in the neutrophil compartment (16, 17).

The above mentioned results triggered the development of highly selective Syk-inhibitors like entospletinib, which seems to have a tolerable safety profile according to clinical trials with hematological malignancies (18, 19). The importance of Syk in autoantibody-mediated inflammation and the human safety data prompted us to test the effect of entospletinib in experimental autoimmune arthritis. Here, we describe that this second generation inhibitor could dose-dependently decrease the clinical signs of inflammatory arthritis in mice without affecting peripheral immune cell numbers and the well-being of the animals. Our results raise the possibility that entospletinib could be a therapeutic option in the treatment of autoimmune arthritis.

## Materials and methods

### Animals

Wild type C57BL/6 mice were purchased from the Hungarian National Institute of Oncology or were obtained from the Institute from Translational Medicine at Semmelweis University. Mice carrying the KRN T cell-receptor transgene were maintained in heterozygous form by mating with C57BL/6 mice (20). NOD mice were purchased from the Jackson Laboratory. Mice were kept in individually sterile ventillated cages (Tecniplast) in a conventional facility. The animal experiments were approved by the Animal Experimentation Review Board of Semmelweis University (Budapest, Hungary) (17).

### Autoantibody-induced experimental arthritis

K/BxN serum transfer arthritis was initiated as previously described (17, 21–24). In short, mice expressing the KRN T cell receptor transgene on the wild type (C57BL/6) genetic background were mated with NOD mice to obtain transgene-positive (arthritic) K/BxN and transgene-negative (non-arthritic) BxN mice. The presence of the transgene was detected by allele-specific PCR and determined by phenotypic assessment. Blood was taken by retroorbital bleeding and sera from arthritic and non-arthritic mice were pooled separately (17). Experimental arthritis was induced in 6-10 week-old C57BL/6 mice by a single intraperitoneal injection of 300 μl K/BxN (arthritic) serum. BxN (control) serum served as control. The severity of arthritis was followed by ankle thickness measurement with a spring-loaded caliper (Kroeplin) and by visible clinical scoring (on a 0-10 scale) by two investigators every day in a conventional animal facility (17, 20). For histological analysis, mice were sacrificed on day 6, their hind limbs were fixed in 4% paraformaldehyde, decalcified, dehydrated, embedded in paraffin, sectioned at 7 μm thickness and stained with hematoxylin and eosin (20).

### Oral administration of the inhibitor

Entospletinib (GS-9973, Selleckchem) was diluted in water and mucilage. Vehicle-treated mice received only water and mucilage. Mice were administered with entospletinib or vehicle orally twice a day by gavage. Vehicle- and entospletinib-treatment started 1 day before arthritis induction.

### Measuring the circulating immune cell numbers and *in vivo* analysis of neutrophil and macrophage accumulation

Peripheral blood was taken from vehicle- or entospletinib-treated mice and anti-Ly-6G and biotinylated anti-CD11b antibodies (clone 1A8 and clone M1/70) plus PerCP-Streptavidin (all from BD Biosciences) were added to the samples. Cells were identified on the basis of the forward/side scatter characteristics and staining by flow cytometry. Cells with Ly-6G- and CD11b-positivity were considered neutrophils, while Ly-6G-negative and CD11b-positive cells were determined as monocytes.

Mice were injected with K/BxN (arthritic) or BxN (control) sera and were treated orally with entospletinib or vehicle twice a day. At the end of the experiment, the mice were sacrificed and the hind and the fore limbs were digested with Liberase (Sigma) (24). The local neutrophil and macrophage accumulation was determined by flow cytometry on the basis of the forward/side scatter characteristics and staining. Cells with Ly-6G and CD11b-positivity were considered neutrophils, while Ly-6G-negative and CD11b-positive cells were determined as macrophages (24).

### *In vivo* analysis of synovial fibroblast (FLS) proliferation and activation

Following hind and fore limb digestion, the local sublining FLS numbers were detected by a CD90.2-PE antibody (clone 30-H12, BD Biosciences). The activation status of FLS was measured by an anti-MHC Class II-FITC antibody (clone M5/114.15.2, Millipore), while intracellular tyrosine-phosphorylation (PY) was detected by an anti-PY antibody (clone PY20, Southern Biotech) after fixation and permeabilization with buffers (Thermo Fischer) by flow cytometry.

### Anti-glucose-6-phosphate isomerase (anti-GPI) antibody ELISA

Blood was collected from arthritic serum-treated mice from the vehicle- or the entospletinib-treated groups on day 6, the samples were centrifuged and the cell-free serum was used for the ELISA. The plate was coated with glucose-6-phosphate isomerase (Cusabio) overnight and the surface was then blocked with 2% goat serum (Gibco) for 1 hour. This was followed by the incubation with the serum samples (diluted in 1: 150) for another 1 hour. After this step, the samples were incubated with anti-mouse-IgG conjugated with HRP (Invitrogen) and after washing, 1-step Ultra TMB (Thermo Fischer) was added. The color reaction was stopped with sulfuric acid and the signal was detected at 450 nm using an automated refractometer.

### Isolation and activation of neutrophils

Mouse neutrophils were isolated from the bone marrow of the femurs and tibias of wild type mice by hypotonic lysis followed by Percoll (GE Healthcare) gradient centrifugation as previously described (23, 24). The cell surface expression of various markers was detected on isolated or circulating neutrophils by anti-Ly6G-PE (clone 1A8), anti-FcγRII/III (clone 2.4G2), anti-FcγRIV (clone 9E9), anti-CD11a (clone M17/4), anti-CD11b (clone M1/70) or anti-CD18 (clone C71/16) antibodies. The antibodies were from BD Biosciences. Where required, primary antibodies were visualized by PerCP-Streptavidin (BD Biosciences). Flow cytometry was performed on a BD FACSCalibur.

Immobilized immune complex (IC) surfaces were obtained by coating the Nunc MaxiSorp F96 plates (Thermo Fisher) with human serum albumin (Sigma) at a 20 µg/ml concentration, followed by blocking and a treatment with a polyclonal anti-human serum albumin antibody (Sigma) at a 1:400 dilution as previously described (25, 26). Integrin-mediated neutrophil cell responses were detected on a fibrinogen (Sigma) surface (at a 150 μg/ml concentration) in the presence or absence of TNF-α (Peprotech) (26).

Neutrophils were incubated with vehicle (DMSO) or different doses of entospletinib for 10 minutes before activation. Neutrophil assays were performed at room temperature. Superoxide release was measured by a cytochrome c reduction assay, cell spreading was followed by phase contrast microscopy, while neutrophils were stimulated for 6 hours for the detection of cytokine release (23, 24).

### Detection of inflammatory mediators

Affected joints were washed out with PBS supplemented with 10 mM EDTA (pH 7.5) and 20 mM HEPES (Sigma). The levels of inflammatory mediators in the supernatants of *in vivo* digested limb samples or of *in vitro* activated neutrophils were measured by commercial ELISA kits (R&D Systems) according to manufacturer’s instructions (23, 24).

### Statistical analysis

Experiments were performed at the indicated number of times. Statistical analysis were carried out by the STATISTICA program using two- or three-way ANOVA (where the inhibitor-, the arthritic-serum-treatment/*in vitro* activation ± time were the independent variables). Graphs and kinetic curves show mean and SEM from all independent *in vitro* experiments or from all individual mice of the different *in vivo* experiments. P-values below 0.05 were considered statistically significant.

## Results

### Entospletinib-treatment reduced the signs of experimental arthritis without causing major health issues

**Figure 1:**
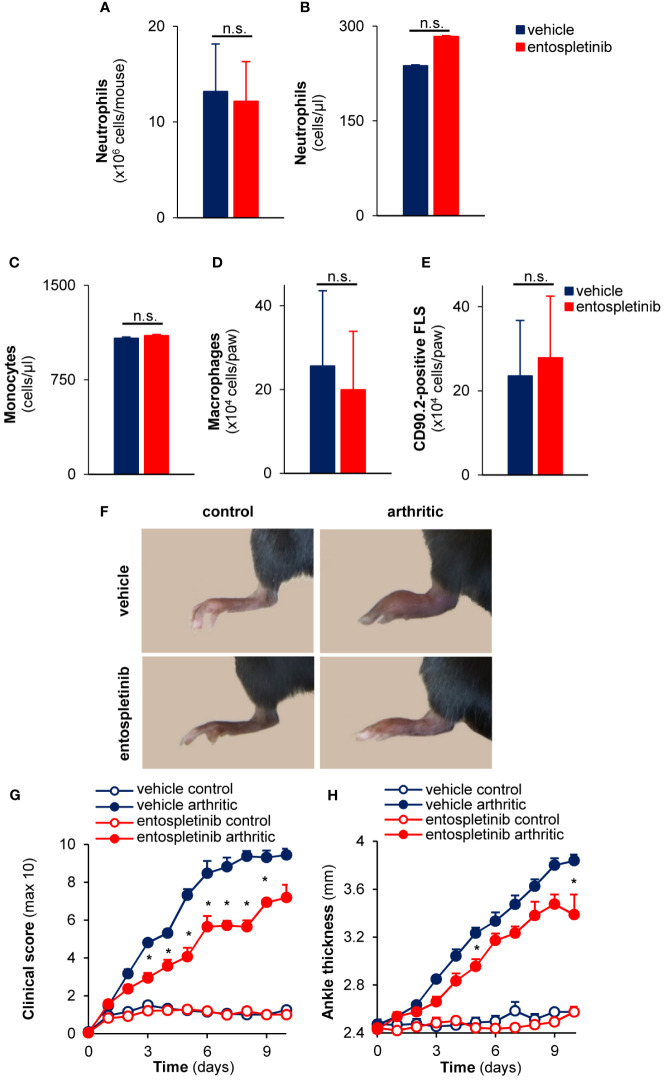
Entospletinib decreased the severity of experimental autoimmune arthritis without influencing bone marrow, circulating or synovial (immune) cell numbers under non-inflammatory conditions. Treatment with the Syk inhibitor did not influence the neutrophil numbers in the bone marrow and in the periheral blood **(A, B)**, while it also did not affect the circulating monocyte and the synovial macrophage or fibroblast counts **(C–E)**. Meanwhile, the administration of 50 mg/kg entospletinib two times a day had a tendency to decrease the severity of the joint inflammation **(F–H)**. Panels **(A–E)** show mean and SEM from 3 independent experiments. Representative images **(F)** or mean and SEM **(G, H)** from 11 control and 13-14 arthritic serum-treated mice in the vehicle or entospletinib group from 5 independent experiments are shown. See the text for actual p values. FLS, synovial fibroblast; n.s., statistically not significant; *p < 0.05.

As neutrophils, macrophages and synovial fibroblasts are important effector cells in the pathogenesis of the anti-GPI antibody-mediated experimental autoimmune arthritis, we tested whether the peripheral or synovial numbers of these cells were affected by the treatment with entospletinib (5, 27–30). We found that neutrophil counts were comparable in the bone marrow and in the peripheral blood in the vehicle- and in the entospletinib-treated groups (**Figures 1A, B**; p = 0.88 and p = 0.89, respectively). In line with these findings, the circulating monocyte or the synovial macrophage and fibroblast numbers also did not show significant differences between the two groups (**Figures 1C–E**; p = 0.99, p = 0.84 and p = 0.45, respectively).

**Figure 2:**
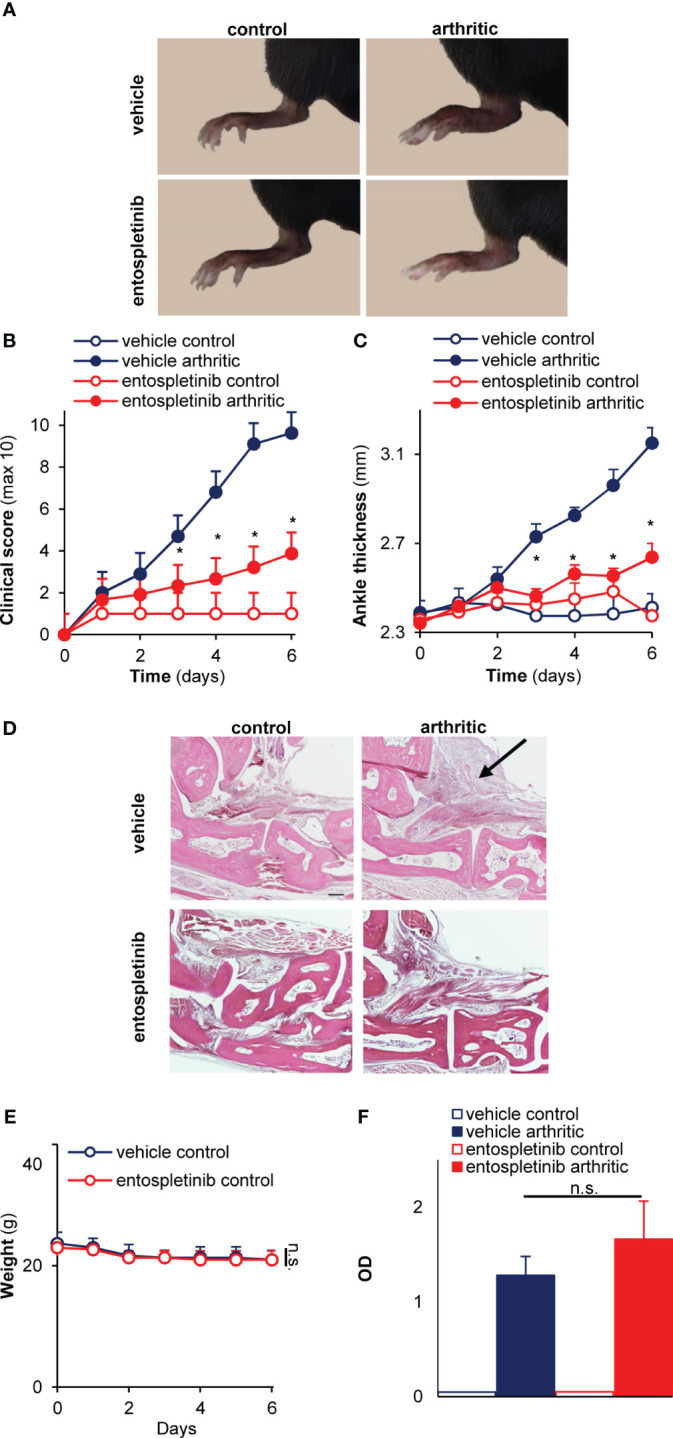
A higher entospletinib dose massively reduced joint inflammation without affecting animal health or the circulating anti-GPI antibody levels. The oral administration of 100 mg/kg entospletinib twice a day massively reduced the severity of experimental arthritis both when we followed the clinical scores or the ankle thickening **(A–C)**. Histological analysis showed a massive leukocyte infiltration in the arthritic serum- and vehicle-treated mice, which was nearly absent from the inhibitor-treated animals **(D)**. Meanwhile, the inhibitor did not influence the weight of the animals during the entire period of the *in vivo* experiment and did not decrease the circulating anti-GPI antibody levels **(E, F)**. Representative images **(A, D)** or mean and SEM **(B,C)** from 3 control and 5-6 arthritic serum-treated mice in the vehicle or the entospletinib group from 3 independent experiments are shown. Panels E and F show mean and SEM from 3 independent experiments. See the text for actual p values. n.s., statistically not significant; *p < 0.05. Scale bar: 200 μm. The arrow points at leukocyte infiltration.

Next, we were interested in whether the inhibitor had any influence on the development and progression of experimental autoimmune arthritis. When treated orally by 50 mg/kg entospletinib twice a day, mice had a reduced inflammatory phenotype compared to the vehicle-treated group: this was detectable on the pictures taken at day 6 or when the clinical scores and the ankle thickness changes were measured (**Figures 1F–H**). The phenotype was even stronger when the animals received 100 mg/kg entospletinib twice a day (**Figures 2A–C**). Histological analysis showed a massive leukocyte infiltration in the arthritic serum- and vehicle-treated mice, which was nearly absent from the inhibitor-treated animals (**Figure 2D**). Meanwhile, the inhibitor did not cause visible signs of health problems or weight loss in the control serum-treated mice compared to the vehicle-consuming animals, pointing at the well-tolerability of entospletinib (**Figure 2E**; p = 0.99). Furthermore, the Syk inhibitor did not trigger a decline in the circulating anti-GPI antibody levels, which means that entospletinib did not interfere with the elimination of the autoantibodies (**Figure 2F**; p = 0.62).

Taken together, entospletinib dose-dependently reduced the inflammatory signs of autoantibody-induced experimental arthritis without affecting the peripheral and the synovial numbers of critical cellular components under non-inflammatory conditions or the level of the pathogenic anti-GPI antibodies in the circulation after arthritis initiation.

### Entospletinib reduced the recruitment of neutrophils to the inflamed joints, but did not affect monocyte accumulation and synovial fibroblast proliferation

**Figure 3:**
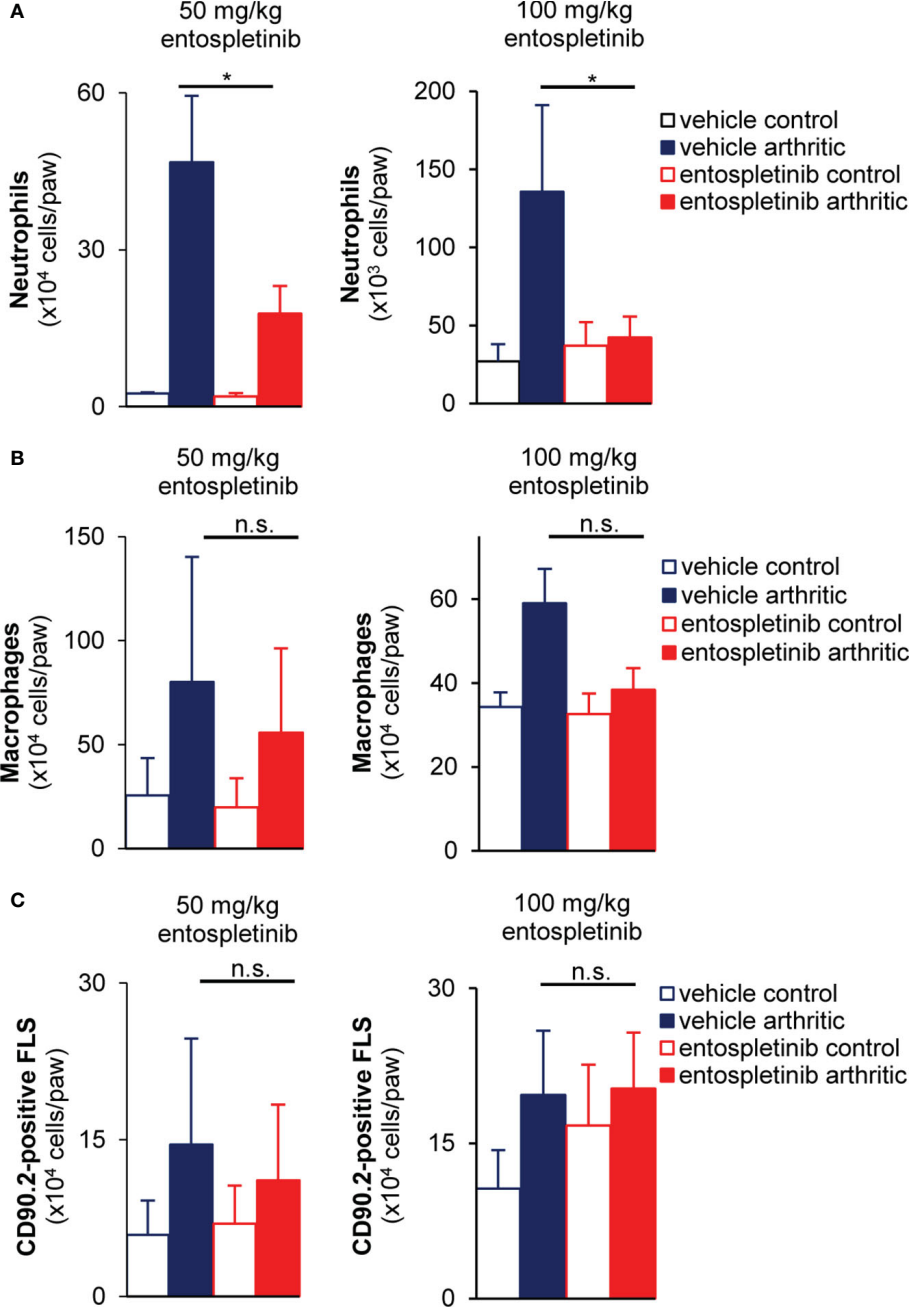
Reduced neutrophil accumulation, but unaffected monocyte recruitment and synovial fibroblast proliferation at the site of inflammation in the presence of the Syk inhibitor. Both doses of entospletinib massively reduced the recruitment of neutrophils to the inflamed joints **(A)**. However, the inhibitor did not have significant effects on the accumulation of monocytes or the proliferation of the immune cell recruiting sublining synovial fibroblasts **(B, C)**. Mean and SEM from 3-5 independent experiments are shown. See the text for actual p values. FLS, synovial fibroblast; n.s., statistically not significant; *p < 0.05.

Next, we examined how entospletinib-treatment affected the recruitment of immune cells. Mice were treated with control or arthritic serum, and they received vehicle or entospletinib by oral gavage. While the inhibitor caused a massive reduction in neutrophil accumulation at the site of inflammation, the recruitment of monocytes was not significantly affected (**Figures 3A, B**; p = 0.048 for the 50 mg/kg and p = 1,9 x 10^-4^ for the 100 mg/kg dose /neutrophils/ and p = 0.07 for the 50 mg/kg and p = 0.065 for the 100 mg/kg dose /macrophages/). In line with the latter, entospletinib-treated mice had similar numbers of the immune cell-recruiting sublining synovial fibroblasts (31) as the vehicle-treated animals (**Figure 3C**; p = 0.45 for the 50 mg/kg and p = 0.42 for the 100 mg/kg dose). Moreover, the activation of synovial fibroblasts, measured by the extent of intracellular tyrosine phosphorylation, did not show significant differences between the entospletinib- and the vehicle treated groups, only a tendency toward reduction (at the higher dose) could be observed (**Supplementary Figure 1A**; p = 0.57 for the 50 mg/kg and p = 0.084 for the 100 mg/kg dose). While the lower dose showed a similar phenomenon in the context of MHC Class II upregulation, the higher inhibitor concentration caused a significant reduction (**Supplementary Figure 1B**; p = 0.99 for the 50 mg/kg and p = 0.034 for the 100 mg/kg dose).

In short, while leaving monocyte recruitment and synovial fibroblast proliferation unchanged, entospletinib effectively reduced the accumulation of polymorphonuclear cells to the synovial area, which could be linked to the macroscopic phenotype.

### Reduced cytokine levels in the inflamed joints in the presence of the Syk inhibitor

**Figure 4:**
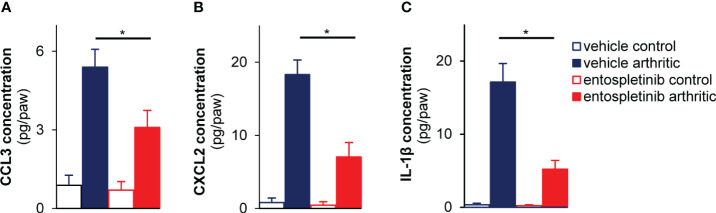
Lower cytokine levels in the affected joints of the entospletinib-treated animals. The Syk inhibitor significantly lowered the investigated cytokine levels in the inflamed joints compared to the vehicle-treated arthritic mice **(A–C)**. Mean and SEM from 3-4 independent experiments are shown. See the text for actual p values. *p < 0.05.

As mouse neutrophils are able to produce cytokines and chemokines upon activation under *in vivo* conditions, we measured the local accumulation of MIP-1α, MIP-2 and IL-1β at the synovial area (24). While arthritic serum-treated mice in the vehicle group had massively increased (partly neutrophil-associated) CCL3 (MIP-1α) and CXCL2 (MIP-2) chemokine levels at the site of inflammation, the oral use of entospletinib could effectively and significantly reduce the concentrations of the cytokines, pointing at an altered inflammatory milieu in the presence of the Syk inhibitor (**Figures 4A, B**; p = 1.6 x 10^-4^ and p = 1.6 x 10^-4^, respectively). Moreover, IL-1β levels were also significantly reduced in the affected joints by the Syk inhibitor (**Figure 4C**; p = 1.6 x 10^-4^). These results with the reduced accumulation of neutrophils raise the possibility that entospletinib mainly (but perhaps not exclusively) exerts its effect on these granulocytes in experimental autoimmune arthritis.

### Decreased neutrophil cell responses on immobilized immune complex surfaces in the presence of entospletinib

**Figure 5:**
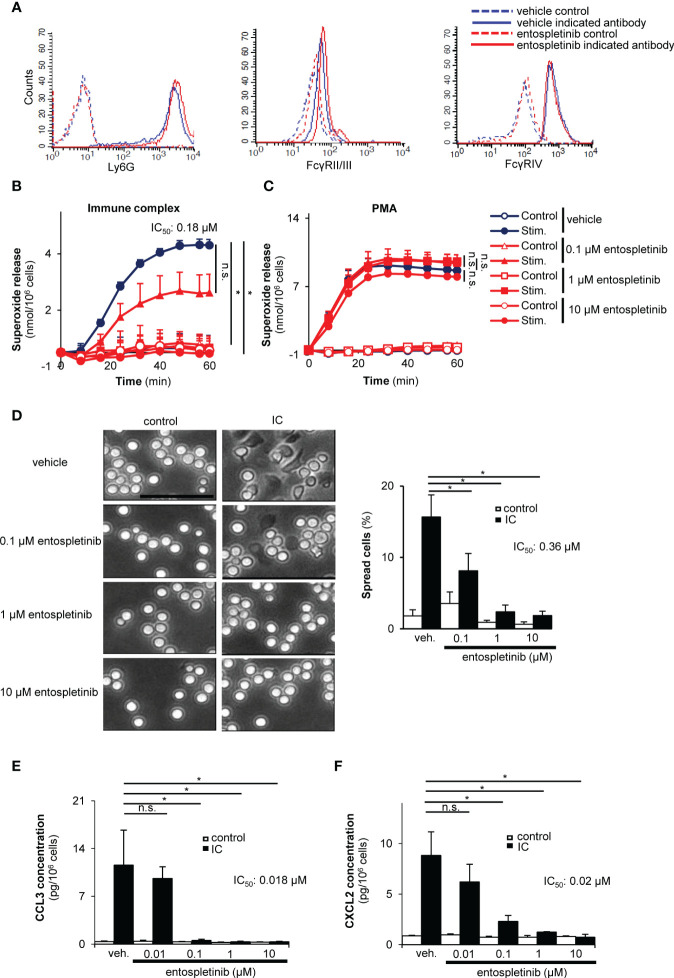
Entospletinib dose-dependenly decreased the immune complex-tiggered *in vitro* neutrophil cell responses. Isolated mouse neutrophils had normal cell surface expression of the maturation marker Ly-6G and the activating Fcγ receptors in the presence of entospletinib **(A)**. When plated on immobilized immune complex surfaces, vehicle-treated neutrophils nicely produced superoxide and spread upon activation, which was dose-dependently reduced by the specific Syk inhibitor **(B, D)**. However, entospletinib did not affect the reactive oxygen species release in the presence of the direct protein kinase C activator PMA, showing that neutrophil functionality was unaffected in the presence of the Syk targeting agent **(C)**. Moreover, the release of the cytokine CCL3 (MIP-1α) and CXCL2 (MIP-2) was dose-dependently reduced by the Syk inhibitor **(E, F)**. Representative images in panels **(A, D)** from 3 independent experiments are shown. (Panels **B**, **C**, **E**, **F**) show mean and SEM from 3 independent experiments. See the text for actual p values. IC, immune complex; n.s., statistically not significant; stim., stimulated; veh., vehicle; *p < 0.05. Scale bar: 50 μm.

Next, we turned our attention to the neutrophil compartment. Entospletinib did not influence the maturation of neutrophils or their cell surface Fcγ receptor expression (**Figure 5A**). In order to model the *in vivo* activation of neutrophils by Fc receptors, we generated immobilized immune complex surfaces *in vitro*. While vehicle-treated freshly isolated mouse neutrophils produced a significant amount of superoxide when plated on immune complex surfaces, entospletinib dose-dependently decreased the release of this toxic oxygen intermediate (**Figure 5B**; p = 0.017 for vehicle vs. 1 µM entospletinib). However, the presence of the inhibitor did not influence the superoxide release tiggered by the allosteric protein kinase C activator PMA, showing that neutrophil functionality was unaltered by entospletinib (**Figure 5C**; p = 0.99 for vehicle vs. 10 µM entospletinib). Furthermore, while vehicle-treated neutrophils spread over the surface upon Fcγ receptor ligation, the presence of the Syk inhibitor reduced the percentage of the spread cells (**Figure 5D**; p = 2.3 x 10^-4^ /vehicle vs. 1 µM entospletinib/ and p = 0.047 /vehicle vs. 0.1 µM entospletinib/). Moreover, entospletinib could nicely decrease not just the short-, but also the long-term cell responses, as its presence reduced the immune complex-triggered production of CCL3 (MIP-1α) and CXCL2 (MIP-2) (**Figures 5E, F**; p = 8.6 x 10^-3^ /vehicle vs. 0.1 µM entospletinib/ and p = 1.1 x 10^-3^ /vehicle vs. 0.1 µM entospletinib/, respectively).

Taken together, entospletinib dose-dependently reduced both the short- and long-term cell responses of Fcγ receptor-stimulated neutrophils, while it did not influence the maturation and the cell surface expression of Fcγ receptors of these granulocytes.

### Entospletinib-treated neutrophils had reduced cell responses when activated through integrins

**Figure 6:**
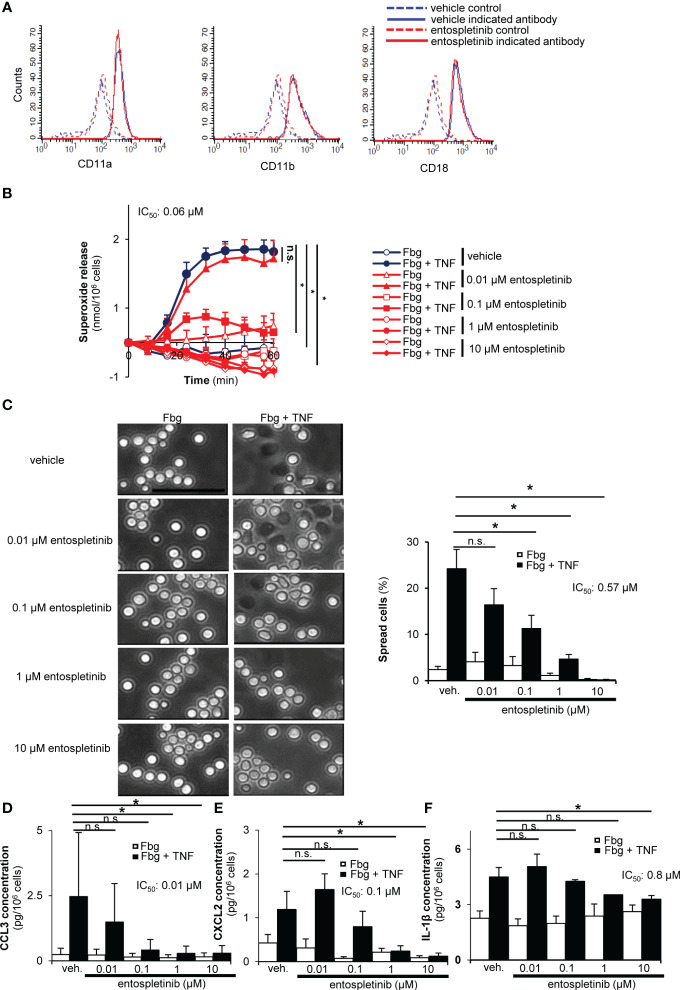
Entospletinib could block the integrin-mediated *in vitro* neutrophil cell responses. Isolated neutrophils had normal cell surface expression of the β_2_ integrin component CD11a, CD11b and CD18 in the presence of entospletinib **(A)**. When plated on fibrinogen in the presence of TNFα, vehicle-treated neutrophils nicely produced superoxide and spread upon activation, which was dose-dependently reduced by the specific Syk inhibitor **(B, C)**. Moreover, the release of the cytokine CCL3, CXCL2 and IL-1β was dose-dependently reduced by entospletinib **(D–F)**. Representative images in panels **(A, C)** from 3 independent experiments are shown. (Panels **B**, **D**–**F**) show mean and SEM from 3-5 independent experiments. See the text for actual p values. Fbg, fibrinogen; n.s., statistically not significant; veh., vehicle; *p < 0.05. Scale bar: 50 μm.

Integrins, especially β_2_ integrins, are important mediators of experimental autoimmune arthritis (4). We found that the Syk inhibitor entospletinib did not affect the cell surface expression of critical β_2_ integrin components like CD11a, CD11b and CD18 (**Figure 6A**). When activated on an integrin ligand surface in the presence of TNF-α, entospletinib dose-dependently reduced the superoxide release and the spreading of the cells compared to the vehicle-treated granulocytes (**Figures 6B, C**; p = 3.5 x 10^-3^ and p = 0.004/vehicle vs. 0.1 µM entospletinib/, respectively). In line with these findings, entospletinib could also attenuate the production of different chemokines like CCL3 and CXCL2 by neutrophils when activated through their β_2_ integrins (**Figures 6D, E**; p = 0.013 and p = 6.6 x 10^-4^/vehicle vs. 1 µM entospletinib/, respectively). Entospletinib also dose-dependently reduced the release of IL-1β (**Figure 6F**; p = 0.029 vehicle vs. 10 µM entospletinib).

These results demonstrated that entospletinib could block the activation of mouse neutrophils when activated by integrin ligands. These observations with those of **Figure 5** indicate that entospletinib is capable of reducing neutrophil functions even when the cells are activated through their Fcγ receptors or their β_2_ integrins, through two important receptors in the pathogenesis of autoimmune arthritis.

## Discussion

Here we report for the first time that a Syk-selective inhibitor could massively and substantially reduce the macroscopic signs of an experimental autoimmune arthritis without causing major health issues in the animals. This exceeds our current knowledge about the effectivity of entospletinib on autoinflammation and graft versus host disease in mice or hematological malignancies in humans (18, 19, 32, 33). Besides decreasing the visible signals of joint inflammation, the second generation Syk-inhibitor entospletinib also lowered the accumulation of neutrophils and the investigated cytokines in the affected joints. Our mechanistic experiments further revealed that this phenomenon could be mimicked by the *in vitro* activation of neutrophils on immune complex or integrin ligand surfaces, where entospletinib dose-dependently decreased all the investigated cell responses (which are required for arthritis development).

The decreased neutrophil recruitment in the presence of entospletinib could be a consequence of an altered migratory capacity of neutrophils. However, entospletinib is a highly selective Syk inhibitor, which interacts with only one other kinase (namely tyrosine kinase non receptor 1 /TNK1/) at a low concentration (18). As TNK1 mainly participates in fetal development, we can presume that entospletinib exerts its inhibitory effect mostly through blocking the activity of the Syk tyrosine kinase. As we and others previously showed that the absence of Syk did not affect the migration of neutrophils under different *in vitro* and *in vivo* settings, the probability that Syk inhibition interferes with cell migration is highly unlikely (10, 34). Instead, the most probable scenario is that entospletinib achieves its inhibitory effect after neutrophils arrived to the site of inflammation by interfering with their Fcγ receptor- and integrin-mediated activation. This would nicely fit into our concept, where the Src family kinase-Syk-CARD9 route, this Fc receptor and integrin signaling axis, mediates tissue inflammation by amplifying the inflammatory process through generating positive feedback loops by neutrophils (23, 24, 34, 35).

The unaltered sublining synovial fibroblast numbers and mainly unaffected activation beside entospletinib treatment can easily contribute to a normal monocyte influx to the inflamed joints (note that not all macrophages in the inflamed synovium derive from the peripheral blood upon arthritic serum treatment as resident macrophages can also be found in the tissue). This process can also initiate neutrophil recruitment according to the concept of Croft and colleagues about the role of sublining FLS in immune cell accumulation (31). It is a probable scenario that neutrophils are partly driven by FLS to the joints, but these granulocytes are unable to carry out their above mentioned positive feedback loops under the influence of entospletinib.

It is an interesting question how entospletinib would affect the activation of other immune cell types than neutrophils. As we used a passive immunization model, the influence of entospletinib on the function of B cells can be excluded. However, when we isolated mononuclear cells from the bone marrow and we investigated the effect of different doses of entospletinib on the basal intracellular tyrosine phosphorylation, we observed a modest decrease in these cells compared to the vehicle-treated group (data not shown). Further analysis revealed that entospletinib could reduce the intracellular tyrosine phosphorylation state of B cells (at a comparable level as in neutrophils), which has translational aspects, as these immune cells are also important in the pathogenesis of rheumatoid arthritis (data not shown) (36). These results raise the possibility that the Syk-selective inhibitor may also influence the function of B cells and the autoantibody production in mouse arthritis models with active immunization (e.g. in the collagen-induced arthritis model). Our results with the robust effect of entospletinib on the neutrophil compartment in this arthritis model may partly rely on the essential role of these granulocytes in the development of joint inflammation.

The maximum serum level that can be reached in entospletinib-treated human individuals is above the range of the half-maximal values of our *in vitro* neutrophil studies (**Figures 5**, **6**), meaning that clinically relevant doses of entospletinib were used in those experiments. In the *in vivo* model, we treated mice with similar doses of entospletinib that were administered in other preclinical experiments (32, 33). Moreover, the orally used 80 mg and 240 mg entospletinib per day per mouse dose in the study of Poe and colleagues resulted in a 1.33 μM and 3.48 μM mean plasma concentration, respectively, which is again above our *in vitro* entospletinib IC_50_ values (33). Taken together, the entospletinib doses used in the *in vitro* and *in vivo* experiments of this study seem to be clinically relevant and help our results to be extrapolated to human investigations. This is strengthened by the fact that entospletinib showed a tolerable safety profile in clinical trials in patients with hematological malignancies (18, 19).

Overall, our results raise the possibility that entospletinib could be used in the treatment of human autoimmune arthritis, maybe alone or as a combination therapy.

## Data availability statement

The original contributions presented in the study are included in the article/**Supplementary Material**. Further inquiries can be directed to the corresponding author.

## Ethics statement

The animal study was approved by the Animal Experimentation Review Board of Semmelweis University (Budapest, Hungary). The study was conducted in accordance with the local legislation and institutional requirements.

## Author contributions

EK: Data curation, Formal Analysis, Investigation, Methodology, Visualization, Writing – original draft. LB: Data curation, Formal Analysis, Investigation, Methodology, Visualization, Writing – original draft. AM: Resources, Writing – review & editing. ÉK: Methodology, Writing - original draft. ZJ: Methodology, Writing – original draft. TN: Methodology, Writing – original draft, Conceptualization, Data curation, Formal Analysis, Funding acquisition, Investigation, Project administration, Resources, Software, Supervision, Validation, Visualization, Writing – review & editing.

## Supplementary material

Supplementary Figure 1
*Entospletinib did not have a major influence on the in vivo activation of sublining synovial fibroblasts* The inhibitor did not have a significant effect on the intracellular tyrosine phosphorylation of FLS **(A)**. While the upregulation of the MHC Class II was unaltered by the lower, we observed a significant reduction with the higher dose of entospletinib **(B)**. Graphs show mean and SEM form 3-5 independent experiments. See the text for actual p values. n.s., statistically not significant; *p < 0.05.
