# Supplementary figures and images for: The selective inhibition of the Syk tyrosine kinase ameliorates experimental autoimmune arthritis
Source: Front Immunol. 2023 Dec 4;14:1279155. doi: 10.3389/fimmu.2023.1279155 (PMC10725968; doi:10.3389/fimmu.2023.1279155)

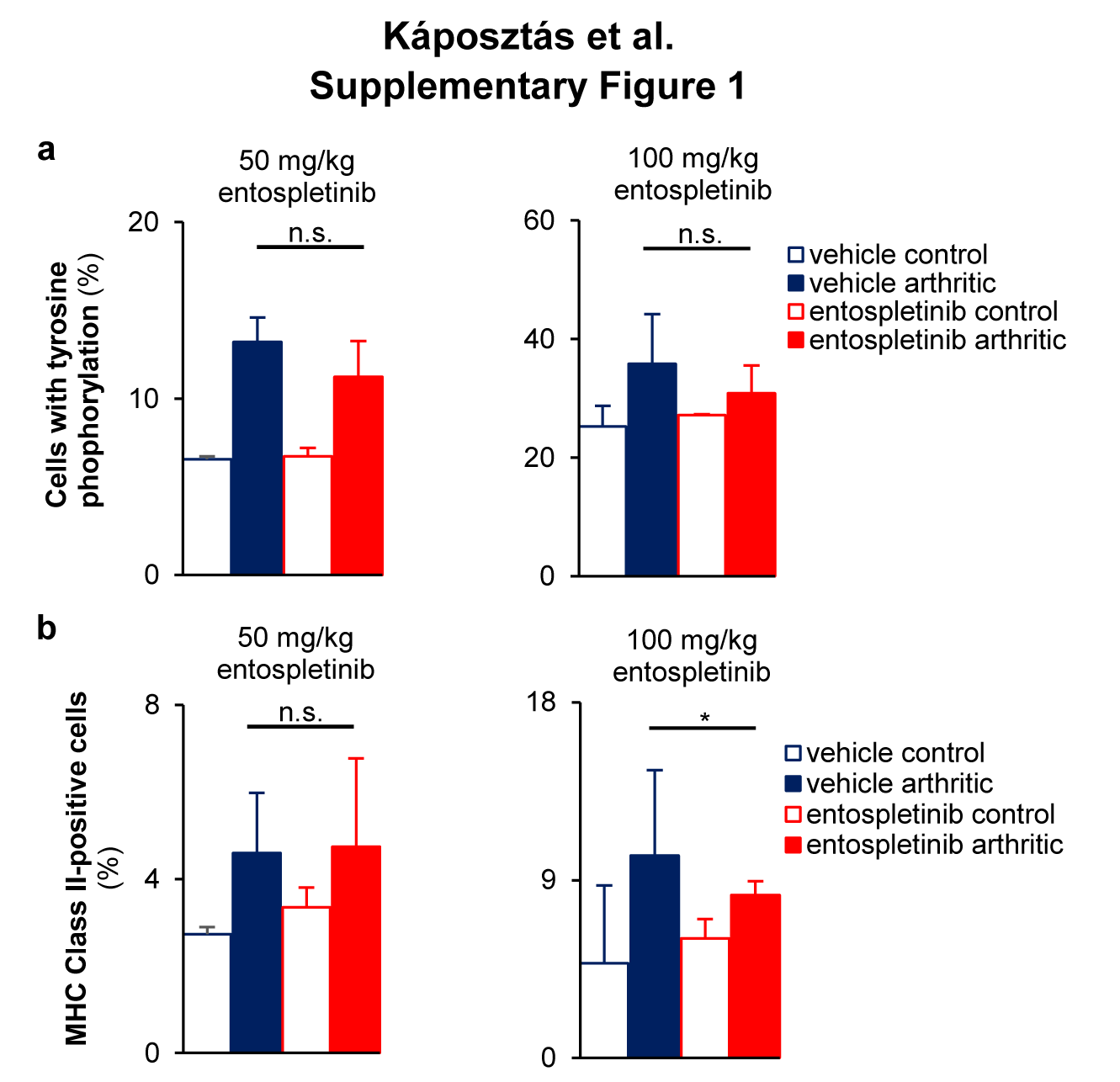

Supplement: Supplementary Figure 1 — Entospletinib did not have a major influence on the in vivo activation of sublining synovial fibroblasts The inhibitor did not have a significant effect on the intracellular tyrosine phosphorylation of FLS (A). While the upregulation of the MHC Class II was unaltered by the lower, we observed a significant reduction with the higher dose of entospletinib (B). Graphs show mean and SEM form 3-5 independent experiments. See the text for actual p values. n.s., statistically not significant; *p < 0.05. [file Image_1.tif]
